# Molecular Basis for Nucleotide Conservation at the Ends of the Dengue Virus Genome

**DOI:** 10.1371/journal.ppat.1002912

**Published:** 2012-09-13

**Authors:** Barbara Selisko, Supanee Potisopon, Rym Agred, Stéphane Priet, Isabelle Varlet, Yann Thillier, Corinne Sallamand, Françoise Debart, Jean-Jacques Vasseur, Bruno Canard

**Affiliations:** 1 Aix-Marseille Université, CNRS, AFMB UMR 7257, 163, Marseille, France; 2 IBMM, UMR 5247 CNRS-UM1-UM2, cc 1704 Université Montpellier 2, Montpellier, France; Washington University School of Medicine, United States of America

## Abstract

The dengue virus (DV) is an important human pathogen from the *Flavivirus* genus, whose genome- and antigenome RNAs start with the strictly conserved sequence pppAG. The RNA-dependent RNA polymerase (RdRp), a product of the NS5 gene, initiates RNA synthesis *de novo*, *i.e.*, without the use of a pre-existing primer. Very little is known about the mechanism of this *de novo* initiation and how conservation of the starting adenosine is achieved. The polymerase domain NS5Pol_DV_ of NS5, upon initiation on viral RNA templates, synthesizes mainly dinucleotide primers that are then elongated in a processive manner. We show here that NS5Pol_DV_ contains a specific priming site for adenosine 5′-triphosphate as the first transcribed nucleotide. Remarkably, in the absence of any RNA template the enzyme is able to selectively synthesize the dinucleotide pppAG when Mn^2+^ is present as catalytic ion. The T794 to A799 priming loop is essential for initiation and provides at least part of the ATP-specific priming site. The H798 loop residue is of central importance for the ATP-specific initiation step. In addition to ATP selection, NS5Pol_DV_ ensures the conservation of the 5′-adenosine by strongly discriminating against viral templates containing an erroneous 3′-end nucleotide in the presence of Mg^2+^. In the presence of Mn^2+^, NS5Pol_DV_ is remarkably able to generate and elongate the correct pppAG primer on these erroneous templates. This can be regarded as a genomic/antigenomic RNA end repair mechanism. These conservational mechanisms, mediated by the polymerase alone, may extend to other RNA virus families having RdRps initiating RNA synthesis *de novo*.

## Author Summary

The 5′- and 3′-ends of RNA virus genomes have evolved towards efficient replication, translation, and escape from defense mechanisms of the host cell. Little is known about how RNA viruses conserve or restore the correct ends of their genomes. The *Flavivirus* genus of positive-strand RNA viruses contains important human pathogens such as yellow fever virus, West Nile virus, Japanese encephalitis virus and dengue virus (DV). The *Flavivirus* genome ends are strictly conserved as 5′-AG…CU-3′. We demonstrate here the primary role of the DV polymerase in the conservation of the first and last genomic residue. We show that DV polymerase contains an ATP-specific priming site, which imposes a strong preference for the *de novo* synthesis of a dinucleotide primer starting with an ATP. Furthermore, the polymerase is able to indirectly correct erroneous sequences by producing the correct primer in the absence of template and on templates containing incorrect nucleotides at the 3′-end. The correct primer is productively elongated on either correct or incorrect templates. Our findings provide a direct demonstration of the implication of a viral RNA polymerase in the conservation and repair of genome ends. Other polymerases from other RNA virus families are likely to employ similar mechanisms.

## Introduction

Most RNA viruses maintain the specific sequences present at the ends of their genomes. The 5′ genome end may carry a cap structure to ensure both genome stability and efficient translation [1]. The 3′-end may carry a poly(A) tail or adopt specific 3′-end sequences required for viral replication [2], [3]. They are generally copied exactly to avoid loss of genetic information, and have supposedly evolved towards optimum replication efficiency. Terminal genome damage can be caused by errors introduced by the viral polymerase during initiation and termination, or by cellular ribonucleases [4]. In addition to special mechanisms to ensure efficient initiation of RNA synthesis, viruses have evolved mechanisms to repair or correct damaged extremities such as the use of abortive transcripts as primers, the generation and use of non-templated primers, and the addition of one or few non-templated nucleotides to the 3′-end by a terminal transferase activity [4]. However, our knowledge about these mechanisms is still very limited. Many RNA virus polymerases, which do not use a primer and thus initiate RNA synthesis *de novo*, generate abortive transcripts during the initiation phase of RNA synthesis [5], [6], [7]. Primer-mediated repair of template extremities was so far only demonstrated for the positive-strand RNA (+RNA) turnip crinkle virus (TCV) [8]. Non-templated primer synthesis by the viral polymerase might be involved in the repair mechanism of TCV [9]. Such mechanism was also proposed as the molecular basis of the reconstitution of 5′-ends of negative-strand RNA (-RNA) respiratory syncytial virus (RSV) replicons [10]. In this study we demonstrate how the dengue virus (DV) RNA-dependent RNA polymerase (RdRp), which starts RNA synthesis *de novo*, plays a decisive role in the nucleotide conservation of viral RNA ends.

DV belongs to the *Flavivirus* genus within the +RNA virus family of *Flaviviridae* together with viruses of the genera *Hepacivirus* and *Pestivirus* [11]. The *Flavivirus* genus comprises around 50 virus species [12] including major human pathogens such as DV, yellow fever virus (YFV), West Nile virus (WNV) and Japanese encephalitis virus (JEV). Flaviviruses harbour the RdRp activity in the C-terminal domain (amino acids 272–900) of non-structural protein NS5 [13], [14], [15], [16], [17]. The N-terminal domain contains methyltransferase activities involved in RNA capping [18], [19]. Evidence has been presented that the N-terminal domain of NS5 also harbours the central RNA capping guanylyltransferase activity [20]. The structure of full-length NS5 is not known but several structures of methyltransferase domains have been determined (for review see [21]). Likewise, crystal structures of *Flavivirus* NS5 RdRp domains have been determined for DV [16] and WNV [22]. All structurally characterized viral RdRps so far adopt the basic fold of the SCOP superfamily of DNA/RNA polymerases. As the other subgroups of this superfamily, DNA-dependent DNA polymerases (DdDp, prototype Klenow fragment of the *E.coli* DdDp I), RNA-dependent DNA polymerase (prototype HIV reverse transcriptase) and DNA-dependent RNA polymerases (DdRp, prototype bacteriophage T7 DdRp), their apo-structure is usually likened to a right hand comprising fingers, palm and thumb subdomains. Viral RdRps contain an encircled active site having connecting elements between the fingers and thumb subdomains. Active sites of viral RdRps performing *de novo* RNA synthesis are additionally closed in their initiation conformation due to the existence of structural elements allowing the stable positioning of the first NTP into a priming site [23], [24]. All *Flaviviridae* RdRps studied so far initiate RNA synthesis *de novo*. Accordingly, *Flavivirus* RdRp domain structures contain a “priming loop” in the thumb subdomain closing the catalytic site [16], [22]. The putative priming loop of DV RdRp was defined as comprising residues 792 to 804. Of particular interest are two aromatic residues near the tip of the loop, W795 and H798, which are conserved in all *Flavivirus* RdRps. They might play the role of an initiation platform to which the base of the priming NTP stacks as it was shown for bacteriophage φ6 [23] and proposed for HCV and BVDV RdRps [25], [26]. Structures of DV RdRp in complex with 3′dGTP as well as two models of *de novo* initiation complexes of DV and WNV RdRps favor Trp795 in the role of the initiation platform [16], [22].

Genomes of *Flaviviridae* lack a poly(A) tail at the 3′-end. A remarkable trait of *Flavivirus* genomes is the strict conservation of the 5′- and 3′-end dinucleotides as 5′ AG…CU 3′. The molecular basis for this strict conservation of the 5′- and 3′-end dinucleotides and/or the use of the same starting nucleotide for +RNA and -RNA strand synthesis by the viral polymerases is not known. Its *Hepacivirus* and *Pestivirus* counterparts have to display higher nucleotide tolerance. They are able to initiate with (A/G)C and G(G/U), respectively, since the 5′- and 3′-ends of *Hepacivirus* genomes of different genotypes correspond to 5′ (A/G)C…GU 3′ and the genomes of pestiviruses to 5′ GU…CC 3′. Interestingly, genomes and antigenomes of non-segmented -RNA (ns-RNA) paramyxoviruses, whose RdRps perform *de novo* RNA synthesis, start with a conserved 5′-AC [10].

Here we show that the strict sequence conservation of *Flavivirus* genome ends is entirely polymerase-encoded. We demonstrate ATP-specific *de novo* initiation using the RdRp domain of DV protein NS5 (NS5Pol_DV_) and specific 10-mer oligonucleotidic RNA templates corresponding to the 3′-end of genomic +RNA and -RNA. We document the existence of a built-in ATP-specific priming site of NS5Pol_DV_. This specific site is one of the means by which NS5Pol_DV_ ensures that the DV genome and antigenome start with an A, the others being several correction mechanisms including the generation of non-templated pppAG primers as well as the preferential formation and elongation of pppAG even on templates with non-cognate 3′-ends. Finally, we show that the ATP-specific priming site is part of the putative priming loop coming from the thumb subdomain. There, residue H798, and not W795, is essential for *de novo* initiation and may act as a priming platform stabilizing the ATP priming nucleotide. DV RdRp is actively involved in the conservation of the correct ends of the genome proving thus a direct example of how RNA viruses maintain the integrity of their genomes. The mechanisms described here may more broadly apply to other RNA viruses having viral RdRps able to initiate RNA synthesis *de novo*.

## Results

### NS5Pol_DV_ generates pppAG by abortive *de novo* initiation on short RNA templates

**Figure 1:**
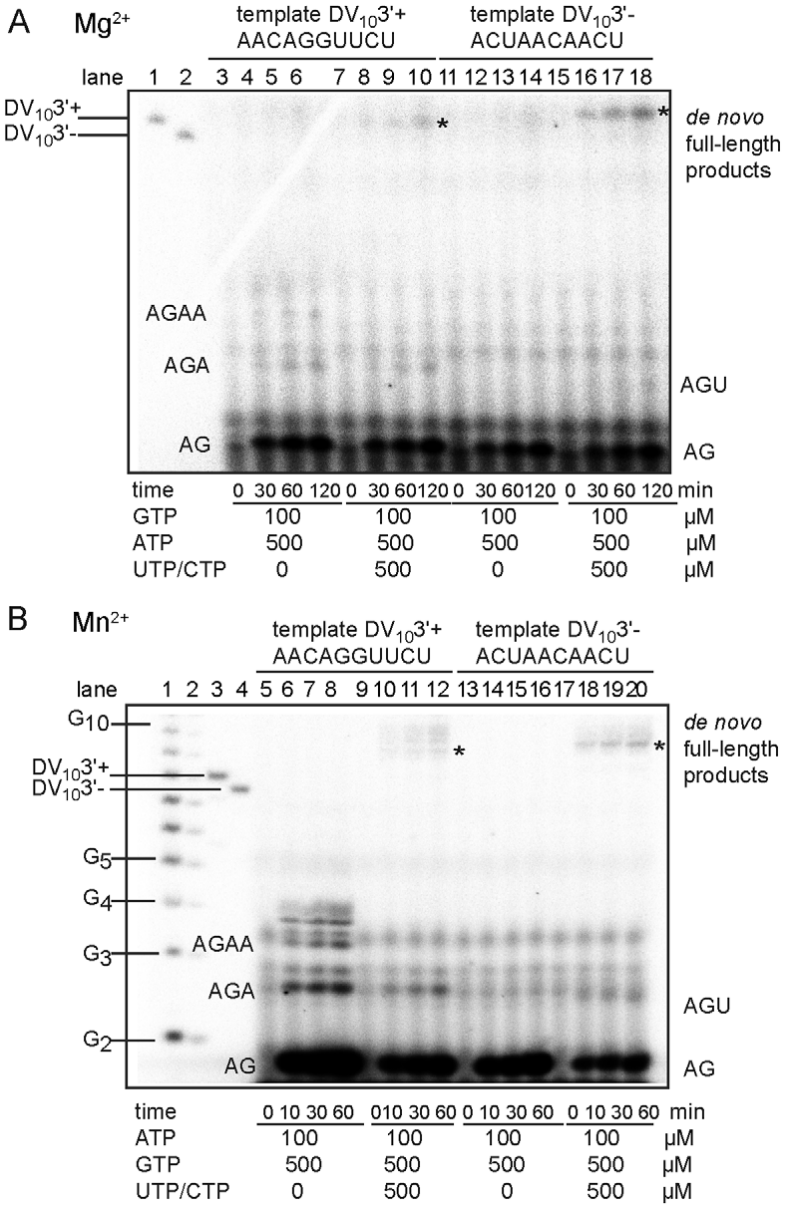
*De novo* initiation by NS5Pol_DV_ on oligonucleotides corresponding to the 3′-ends of dengue virus genome and antigenome. (**A**) *De novo* initiation in the presence of Mg^2+^ ions. Reaction mixtures were prepared as described in Materials and Methods plus 5 mM MgCl_2_, 10 µM template, 500 nM NS5Pol_DV_ and the given concentrations of NTPs. Radiolabeled GTP (αGTP) was used. Reactions were started by the addition of MgCl_2_ and reactions incubated for indicated time periods. Samples were analyzed by PAGE and autoradiography. Markers in lanes 1 and 2 are labeled DV_10_3′+ and DV_10_3′-, respectively. DV_10_3′+ (lanes 3 to 10) and DV_10_3′- (lanes 11 to 18) were used as templates. Nucleotide sequences are given above the panel. Identities of labeled product bands are given on the right and left side of the gel. Full-length products are labeled by an asterisk. (**B**) *De novo* initiation in the presence of Mn^2+^ ions. Reaction mixtures were prepared as indicated in Materials and Methods plus 5 mM MgCl_2_, 2 mM MnCl_2_, 1 µM template, 500 nM NS5Pol_DV_ and the given concentrations of NTPs. Radiolabeled ATP (γATP) was used. Reactions were started by the addition of MnCl_2_, incubated for given time periods, and analyzed by PAGE and autoradiography. Markers in lanes 1 to 4 include an oligoG-ladder (lanes 1 and 2), labeled DV_10_3′+ (lane 3) and DV_10_3′- (lane 4). DV_10_3′+ (lanes 5 to 12) and DV_10_3′- (lanes 13 to 20) were used as templates. Identities of labeled product bands are given on the right and left side of the reaction lanes. Full-length products are labeled by an asterisk.

We set out to study primer synthesis by the RdRp domain of dengue virus protein NS5 (NS5Pol_DV_) using small specific templates corresponding to the 3′-ends of the genome (+RNA) and the antigenome (-RNA). Templates are comprised of 10 nucleotides and are predicted to be devoid of stable secondary structure (see Materials and Methods). Both templates end with the dinucleotide 5′-CU-3′. Product formation over time was followed using either ATP and GTP, or all NTPs needed to form a full-length product when synthesis is precisely started at the 3′-end of the template. Figure 1 shows reaction kinetics of RNA synthesis on DV_10_3′+ corresponding to the 3′-end of the RNA genome 5′-AACAGGUUCU-3′ (left) and on DV_10_3′- corresponding to that of the antigenome 5′-ACUAACAACU-3′ (right). We used either [α-^32^P]-GTP (αGTP, panel A) or [γ-^32^P]-ATP (γATP, panel B) as the radioactive nucleotide. For the catalytic ion, either Mg^2+^ (panel A) or Mg^2+^ supplemented with Mn^2+^ (panel B) were used at their optimum concentrations 5 mM for Mg^2+^ and 2 mM for Mn^2+^ [14]. Reactions with ATP and GTP render time-dependent accumulation of a short product migrating below the marker G2 (see panel B). Comparison with authentic unlabeled pppAG (see Materials and Methods) visualized using UV-shadowing indicated that it indeed corresponds to pppAG (not shown), the expected product of the first step of *de novo* RNA synthesis. When DV_10_3′+ is used as a template, pppAG is formed as well as pppAGA and pppAGAA. When all NTPs are used, pppAG accumulates with time as does pppAGA in the case of DV_10_3′+ and pppAGU in the case of DV_10_3′-. After the synthesis of trinucleotides NSPol_DV_ adopts a processive RNA synthesis elongation mode to continue synthesis up to full-length products (labeled by asterisks in Figure 1). As we had observed before [14], when using Mn^2+^ the reaction is much more efficient and allows for the use of [γ-^32^P]-ATP (γATP) as radiolabeled nucleotide in order to visualize exclusively *de novo* RNA synthesis products starting with ATP. The pattern observed with Mg^2+^ is reproduced when Mn^2+^ is present (Figure 1B). One difference is that the use of Mn^2+^ results in longer full-length products, which might be caused by an alteration of the terminal nucleotide transferase activity of NS5Pol_DV_ [14], [27], [28].

In conclusion, using RNA templates mimicking viral sequences, dinucleotide and trinucleotide products are formed during initiation and before processive RNA elongation, the most abundant being the dinucleotide pppAG.

### NS5Pol_DV_ contains a built-in ATP-specific priming site for *de novo* RNA synthesis initiation

**Figure 2:**
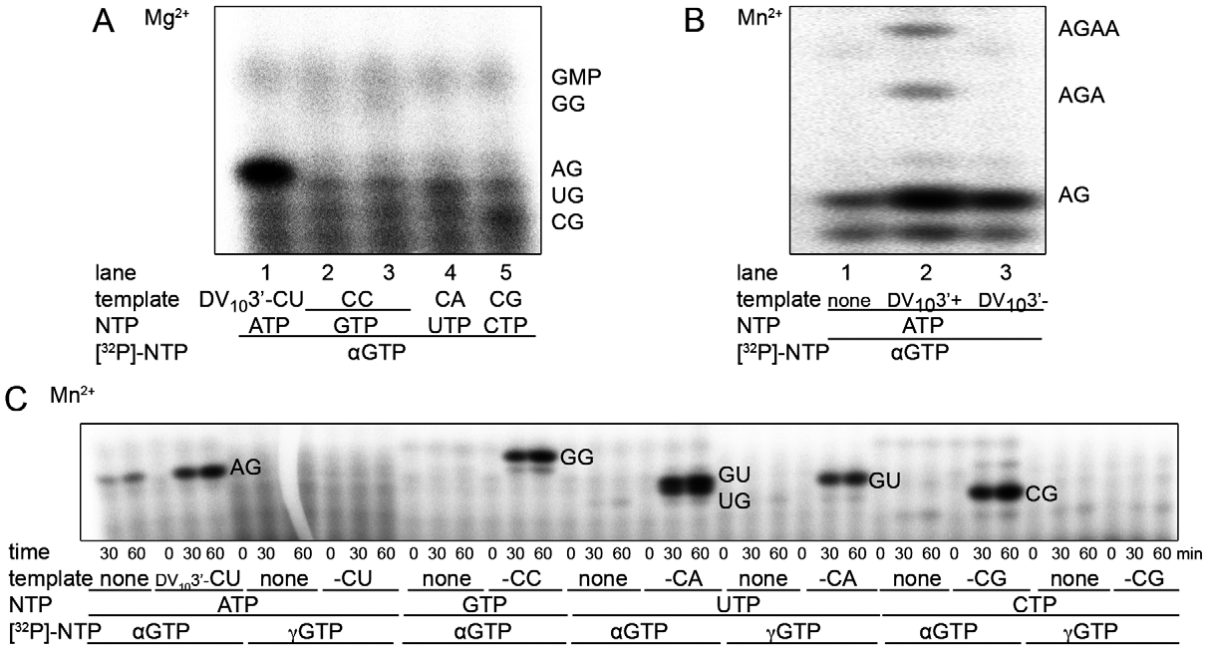
Specificity for ATP as the initiating nucleotide. (**A**) Specific pppAG dinucleotide formation by NS5Pol_DV_ in the presence of Mg^2+^ on DV_10_3′- templates (ACUAACAA-CU) with varying last nucleotides: lane 1 -CU, lanes 2 and 3 -CC, lane 4 -CA and lane 5 -CG) in presence of Mg^2+^. Corresponding initiating NTPs and GTP were used as substrates. Reaction mixtures were prepared as given in Materials and Methods plus 5 mM MgCl_2_, 500 nM NS5Pol_DV_, 10 µM template, 500 µM of initiating NTPs, and 100 µM GTP (containing αGTP). For the reaction on the -CC template, 300 µM (lane 2) and 600 µM GTP (lane 3) was used. Reactions were started by the addition of MgCl_2_ and incubated for 2 h. Samples were analyzed using PAGE and autoradiography. (**B**) pppAG dinucleotide formation by NS5Pol_DV_ in the presence of Mn^2+^. Reaction mixtures contained 2 mM MnCl_2_, 500 nM NS5Pol_DV_, 500 µM GTP, and 100 µM ATP (containing αATP) and either no template (lane 1), 1 µM DV_10_3′+ (lane 2), or 1 µM DV_10_3′- (lane 3). Reactions were started by the addition of MnCl_2_ and incubated for 2 h. The identity of product bands is given on the right. (**C**) Specific non-templated pppAG dinucleotide formation and non-specific NG dinucleotide formation on DV_10_3′- template variants (see under **A**) in the presence of Mn^2+^. Reaction mixtures contained 2 mM MnCl_2_, 500 nM NS5Pol_DV_, 1 µM template, 500 µM of NTPs, which were not labeled, and 100 µM GTP (containing either αGTP or γGTP as outlined below the gel) and either no template or DV_10_3′- variants (given below the gel). Reactions were started by the addition MnCl_2_ and samples were taken at given time points. The identity of product bands is given on the right side of the reaction kinetics.

The first nucleotide of *Flavivirus* genomes is an adenosine, followed by a guanosine. This 5′-pppAG sequence is strictly conserved along the *Flavivirus* genus. In order to answer the question whether the polymerase (and/or the correct template) is at the origin of the conservation of the first nucleotide, we tested a set of DV_10_3′- variants with different 3′-ends. In addition to the correct DV_10_3′- CU, we used DV_10_3′- CC, DV_10_3′- CA and DV_10_3′- CG in the presence of the corresponding priming NTP and GTP. The expected primer products are pppAG, pppGG, pppUG and pppCG, respectively. Figure 2A compares end points of reactions performed in the presence of αGTP and Mg^2+^ as the catalytic ion. Remarkably, the CU template only is proficient for product synthesis (pppAG). RNA primer synthesis on other templates is almost undetectable. We conclude that in the presence of Mg^2+^ as a catalytic ion the DV RdRp priming-site accommodates exclusively ATP.

**Figure 3:**
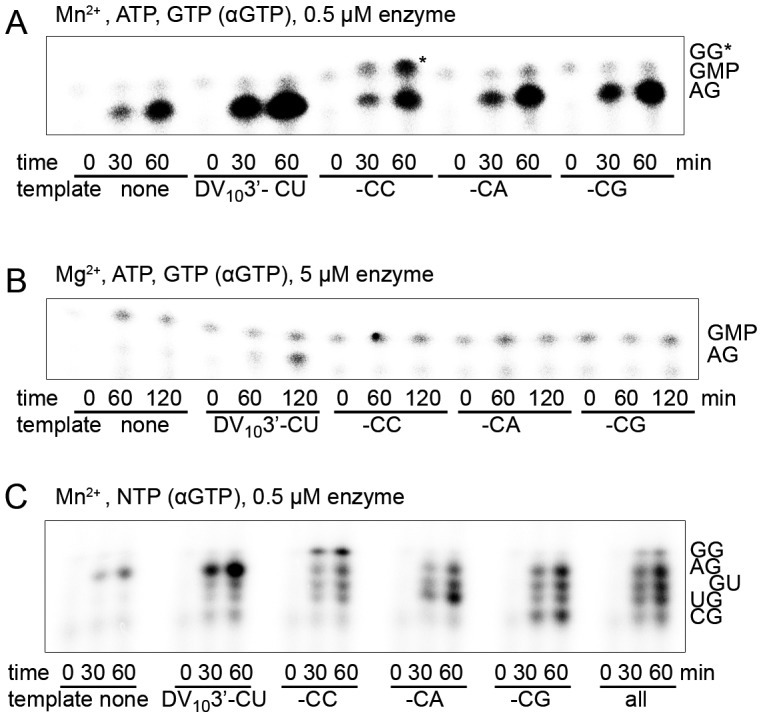
pppAG-formation on the correct DV_10_3′- and on variant templates having an incorrect last nucleotide. Dinucleotide formation by NS5Pol_DV_ on DV_10_3′- templates (ACUAACAA-CN) varying the last nucleotide (correct -CU versus -CC, -CA and -CG). Control reactions were included without template. (**A**) pppAG dinucleotide formation in the presence of Mn^2+^. Only ATP (500 µM) and GTP (100 µM containing αGTP) were used as substrates. Reaction mixtures were prepared as given in Materials and Methods plus 2 mM MnCl_2_, 500 nM NS5Pol_DV_, and 1 µM template. Reactions were started by the addition of MnCl_2_ and samples were taken at given time points. Samples were analyzed by PAGE and autoradiography. pppGG is marked by an asterisk for clarity. (**B**) pppAG dinucleotide formation in the presence of Mg^2+^. Only ATP (500 µM) and GTP (100 µM containing αGTP) were used as substrates. Reaction mixtures were prepared as given in Materials and Methods plus 5 mM MgCl_2_, 5 µM NS5Pol_DV_, and 1 µM template. Reactions were started by addition of MgCl_2_ and samples were taken at given time points. Samples were analyzed as under A. (**C**) pppNG dinucleotide formation in the presence of Mn^2+^. All NTPs were given as substrates at equal concentration (100 µM). Reaction mixtures were prepared as given in Materials and Methods plus 2 mM MnCl_2_, 500 nM NS5Pol_DV_, and 1 µM template. 1 µM overall RNA concentration was used when all templates were present. Radiolabelled αGTP was used. Reactions were started by addition of MnCl_2_ and samples were taken at given time points and analyzed as in A.

To our surprise, when Mn^2+^ was used instead of Mg^2+^, the pppAG primer was generated even in the absence of the template, albeit to a lower extent (Figure 2B). This is not the case in the presence of Mg^2+^ even at ten-fold higher enzyme concentration (see below Figure 3B). When using Mn^2+^ and the DV_10_3′- template variants, we therefore included control reactions in the absence of corresponding templates and in the presence of γGTP, which allows exclusive detection of dinucleotides starting with pppG. Figure 2C shows corresponding reaction kinetics with Mn^2+^ as the catalytic ion in the absence or the presence of templates using αGTP or γGTP as the radioactive nucleotide. Again, using DV_10_3′-CU and ATP/GTP, NS5Pol_DV_ generates pppAG to a higher extent than without template. Note that no pppGA product is generated. When DV_10_3′-CC and GTP is used, NS5Pol_DV_ synthesizes pppGG in the presence of the template only. DV_10_3′-CA, UTP, and GTP lead to the formation of pppUG and pppGU (see γGTP control reaction), the latter by initiation internal to the template. No product is formed in the absence of the template. Finally, DV_10_3′-CG allows formation of pppCG which is not formed in the absence of the template. In conclusion, NS5Pol_DV_ keeps the strict preference for an ATP as the priming nucleotide in the presence of Mn^2+^ when no template is present. Nevertheless, the use of templates with an altered 3′-nucleotide can force NS5Pol_DV_ to start the *de novo* RNA synthesis with the corresponding base-paired priming nucleotide, and also allows internal initiation.

Collectively, these observations confirm that the priming site of NS5Pol_DV_ has a marked specificity for ATP. This preference is strict in the presence of Mg^2+^. It is equally strict for dinucleotide synthesis in the presence of Mn^2+^ and in the absence of template. The specificity for ATP as the starting nucleotide is lost when Mn^2+^ is used in the presence of templates with incorrect 3′-ends; only then NS5Pol_DV_ is able to form pppNG products as efficiently as pppAG.

### The ATP-specific priming site enables NS5Pol_DV_ to produce and elongate the correct primer pppAG on viral templates with non-canonical 3′-nucleotides

In the presence of Mg^2+^ and/or Mn^2+^ the built-in ATP-specific priming site drives NS5Pol_DV_-mediated RNA synthesis starting with pppA. The dinucleotide pppAG is accumulated during RNA synthesis on templates with the correct 3′-end (see Figure 1). Using Mn^2+^ this pppAG primer is also formed in the absence of an RNA template. We asked the question whether NS5Pol_DV_ forms and/or elongates pppAG even on templates with incorrect 3′-nucleotides thus enabling to repair incorrect 3′-ends.

First, pppAG formation was tested on the four DV_10_3′- variants in the presence of only ATP and GTP. Figure 3A shows that NS5Pol_DV_ is indeed able to form pppAG in the presence of templates with any 3′-nucleotide and Mn^2+^. In contrast, in the presence of Mg^2+^ only the natural DV_10_3′- CU template supports pppAG formation even in the presence of an increased concentration of NS5Pol_DV_ (Figure 3B). We then tested pppAG formation exclusively in the presence of Mn^2+^ on all DV_10_3′- variants in the presence of all nucleotides, a scenario putatively mimicking the situation within the replication complex. Figure 3C shows that pppAG is always formed in parallel to the dinucleotide, which corresponds to the template. In the case of the template variant with a -CG 3′-end, pppAG is produced with even higher efficiency than the base-paired dinucleotide. Note that the dinucleotide pppGU is also produced on all templates by internal initiation. For the reaction in the presence of all templates and all nucleotides, we quantified all products, which were initiated *de novo* over the very 3′-end, and found that pppAG is formed as the prominent product (32.3±1.5%, three independent reactions). Note that all templates are present at the same concentration, which should not correspond to the situation *in vivo*. We conclude that in the presence of incorrect templates and Mg^2+^, NS5Pol_DV_ discriminates against these templates and forms pppAG only on the correct template (see also Figure 2A). In contrast, Mn^2+^ ions enable NS5Pol_DV_ to preferentially generate pppAG even in the presence of incorrect templates, which could represent an indirect way of 3′-end repair.

**Figure 4:**
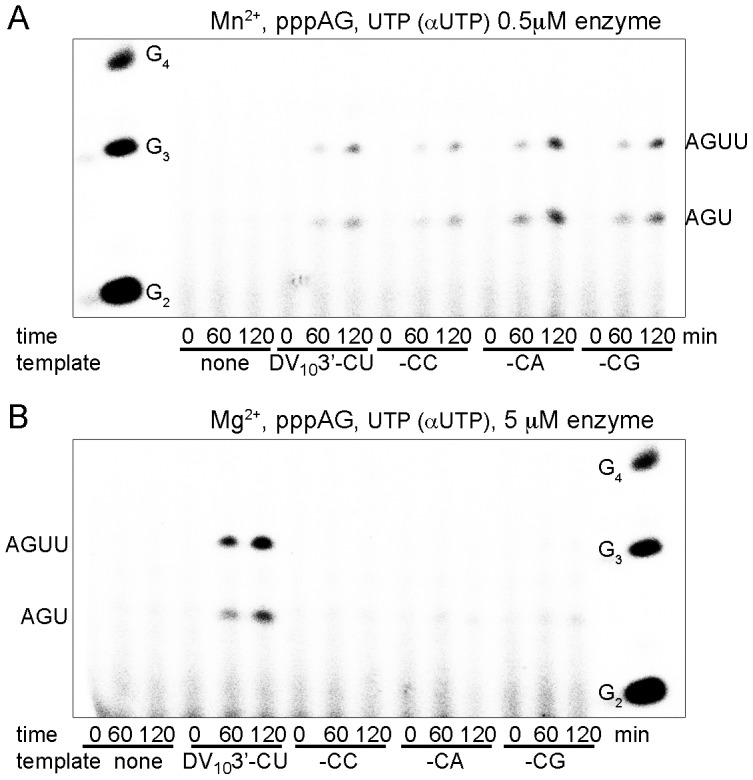
pppAG-elongation on the correct antigenome 3′-end and on variants with an incorrect last nucleotide. pppAG-elongation by NS5Pol_DV_ on DV_10_3′- templates (ACUAACAA-CN) varying the last nucleotide (correct -CU versus -CC, -CA and -CG). Control reactions were included without template. (**A**) pppAG elongation in the presence of Mn^2+^. pppAG (100 µM) and UTP (100 µM, containing αUTP) were used as substrates. Reaction mixtures were prepared as given in Materials and Methods plus 2 mM MnCl_2_, 500 nM NS5Pol_DV_, and 1 µM template. Reactions were started by addition of MnCl_2_ and UTP. Samples were taken at given time points and analyzed by PAGE and autoradiography. OligoG marker is shown on the left, the identity of product bands is given on the right. (**B**) pppAG-elongation in the presence of Mg^2+^. pppAG (100 µM) and UTP (100 µM, containing αUTP) were used as substrates. Reaction mixtures were prepared as given in Materials and Methods plus 5 mM MgCl_2_, 5 µM NS5Pol_DV_, and 1 µM template. Reactions were started by addition of MgCl_2_ and UTP. Samples were taken at given time points and analyzed by PAGE and autoradiography. OligoG marker is shown on the right; the identity of product bands is given on the left.

We then considered the elongation of the correct pppAG primer over templates with incorrect 3′-ends. We thus tested the elongation of a chemically synthesized pppAG primer (see Materials and Methods) either without template or in the presence of the four DV_10_3′- variants (Figure 4). The most prominent result is that NS5Pol_DV_ is able to productively elongate pppAG on the correct template in the presence of Mn^2+^ (Figure 4A) and Mg^2+^ ions (Figure 4B). We also observe that NS5Pol_DV_ in the presence of Mn^2+^ is able to productively elongate pppAG on incorrect templates (Figure 4A), thus demonstrating that the enzyme is able to indirectly correct the error in the template and conserve the 5′-end of the DV genome. Note that as expected there is no primer elongation detectable in the absence of a template.

### The predicted T794-A799 priming-loop of NS5Pol_DV_ provides the built-in ATP-specific priming site

**Figure 5:**
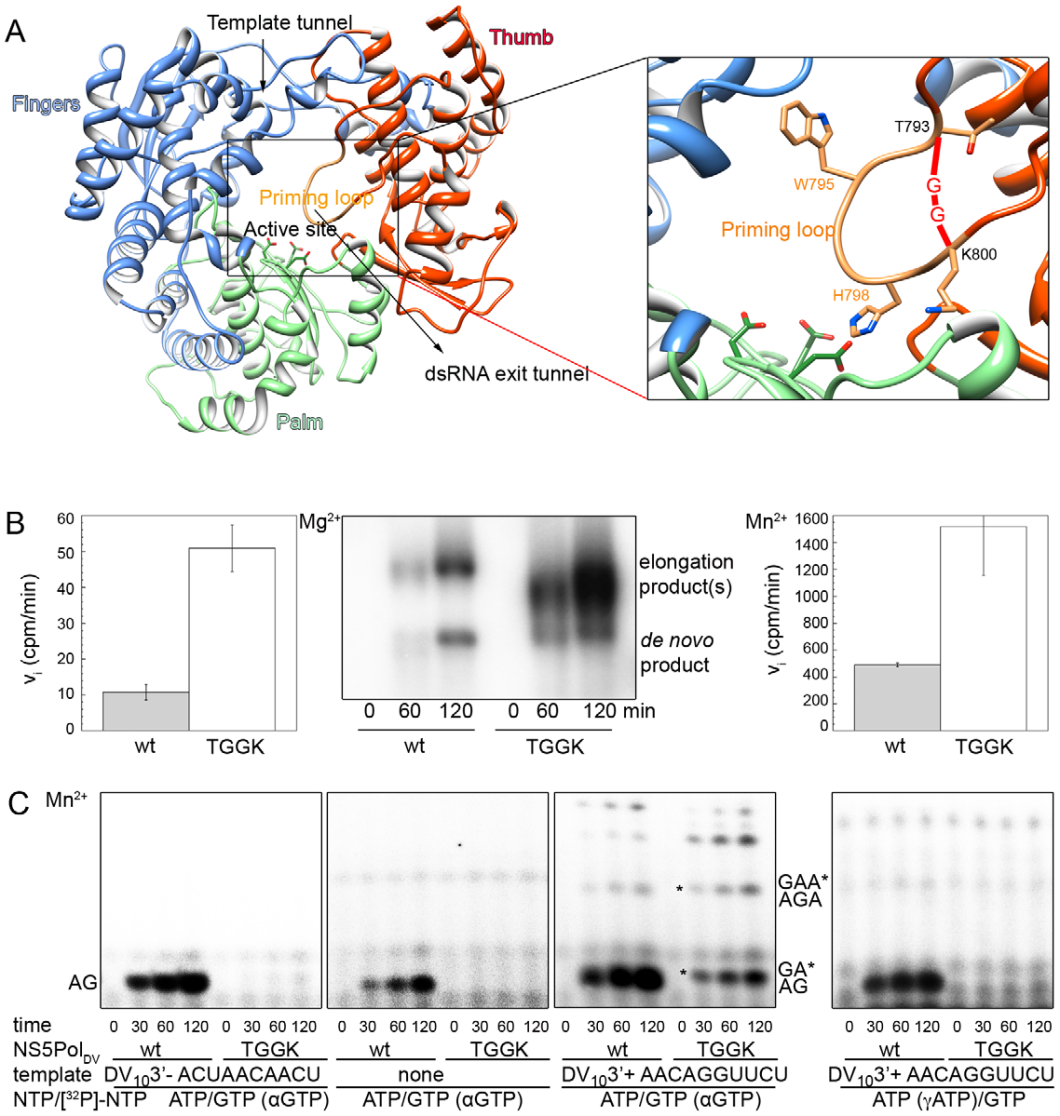
Role of the predicted priming loop T794-A799 in correct *de novo* initiation. (**A**) 3D-structural model of NS5Pol_DV_ used in this study (DV serotype 2 strain New Guinea C) derived from the structure of serotype 3 NS5Pol_DV_ (PDB code 2J7W [16]). NS5Pol_DV_ adopts the typical closed right-hand structure of RdRps containing the palm (light green), fingers (light blue) and thumb (red) subdomains. Between fingers and thumb subdomains the template tunnel runs down to the active site harbored mainly by the palm subdomain. The side chains of the three conserved catalytic residues D533 (motif A), D663 and D664 (motif C) in the active site are shown in sticks (C-atoms light green, O-atoms red). The priming loop emerges from the thumb subdomain and closes the dsRNA exit tunnel and the active site. The close-up shows aromatic residues W795 and H798 (in sticks) within the putative priming loop T794 to A799. In the mutant TGGK the priming loop was replaced by two glycines situated between T793 and K800 (in sticks). (**B**) Activity of wt NS5Pol_DV_ and its deletion mutant TGGK NS5Pol_DV_ was determined on a specific minigenomic template. Reaction mixtures were prepared as given in Materials and Methods. Initial velocities in cpm/min determined by filter-binding assays in the presence of [^3^H]-UTP and liquid scintillation counting, are compared in the presence of Mg^2+^ (left panel) and Mn^2+^ (right panel). The center panel shows agarose-formaldehyde gel analysis of reaction kinetics in the presence of [α-^32^P]-UTP and Mg^2+^ ions. Product bands are labeled on the right sight of the gel. (**C**) *De novo* initiation of wt NS5Pol_DV_ and its deletion mutant TGGK was followed in the presence of Mn^2+^ using either 1 µM DV_10_3′-, in the absence of a template, or 1 µM DV_10_3′+ (from left to right as indicated). Reaction mixtures also contained 2 mM MnCl_2_, 500 nM enzyme, 500 µM of NTPs, which were not labeled, and 100 µM labeled NTP (containing αGTP or γATP as indicated). Reactions were started by addition of MnCl_2_ and samples were taken at given time points. Identities of labeled product bands are given on the right and left side of the reaction kinetics. pppGA and pppGAA internal *de novo* initiation products on DV_10_3′+ are labeled by an asterisk.

NS5Pol_DV_ harbors an ATP-specific priming site, which is essential for the formation, accumulation, and elongation of the correct primer pppAG. Which elements of NS5Pol_DV_ form this site? The crystal structure of NS5Pol_DV_ (Figure 5A) allowed the prediction of a priming loop comprising residues 792 to 804 [16], which is expected to provide the priming site during *de novo* RNA synthesis initiation. We generated a deletion mutant (NS5Pol_DV_ TGGK) by replacing residues T794-A799 between T793 and K800 by two glycines (see close-up in Figure 5A). The overall correct folding of the purified, recombinant mutant protein was verified by a fluorescent thermal shift assay giving identical temperatures of denaturation (melting temperature T_m_) for both proteins (wild type (wt) NS5Pol_DV_ T_m_ 49.0°C ± 0.5°C, NS5Pol_DV_ TGGK T_m_ 48.4°C ± 0.05°C).

The TGGK mutant is expected to have an open active site, which impedes correct ATP-specific *de novo* initiation over the 3′-end of a single-stranded RNA template but may favor the accommodation of double-stranded RNA. Its RNA synthesis initiation and elongation activity was first tested using a “minigenomic” RNA template consisting of 224 nucleotides of the 5′-end of the DV genome fused to 492 nucleotides of the 3′-end [14]. It has been shown before using this template and analyzing the products on a denaturing agarose-formaldehyde gel [29] that two types of product are formed (see wt reaction kinetics in the center panel of Figure 5B). Firstly, the *de novo* RNA synthesis product is generated corresponding to the size of the template. Secondly, an elongation product is generated by back-primed RNA synthesis. There, the 3′-end (…AACAGGUUCU-3′) forms a short hairpin annealing the last di-nucleotide to nucleotides -6 and -7 (underlined in the sequence) and is then elongated [29]. The length of the product is thus ∼twice the size of the template. Reactions were carried out using either Mg^2+^ or Mn^2+^ as catalytic ions. The left and right panels of Figure 5B show that in both cases the mutant TGGK shows an increased overall activity on this template compared to wt activity. The center panel shows that this is mainly caused by increased back-priming. Interestingly, instead of one product species of twice the template size NS5Pol_DV_ TGGK produces a range of elongated products of different lengths. This might be due to the accommodation of long hairpins, which then create longer products than the template but shorter than the elongation product of wt NS5Pol_DV_.

**Figure 6:**
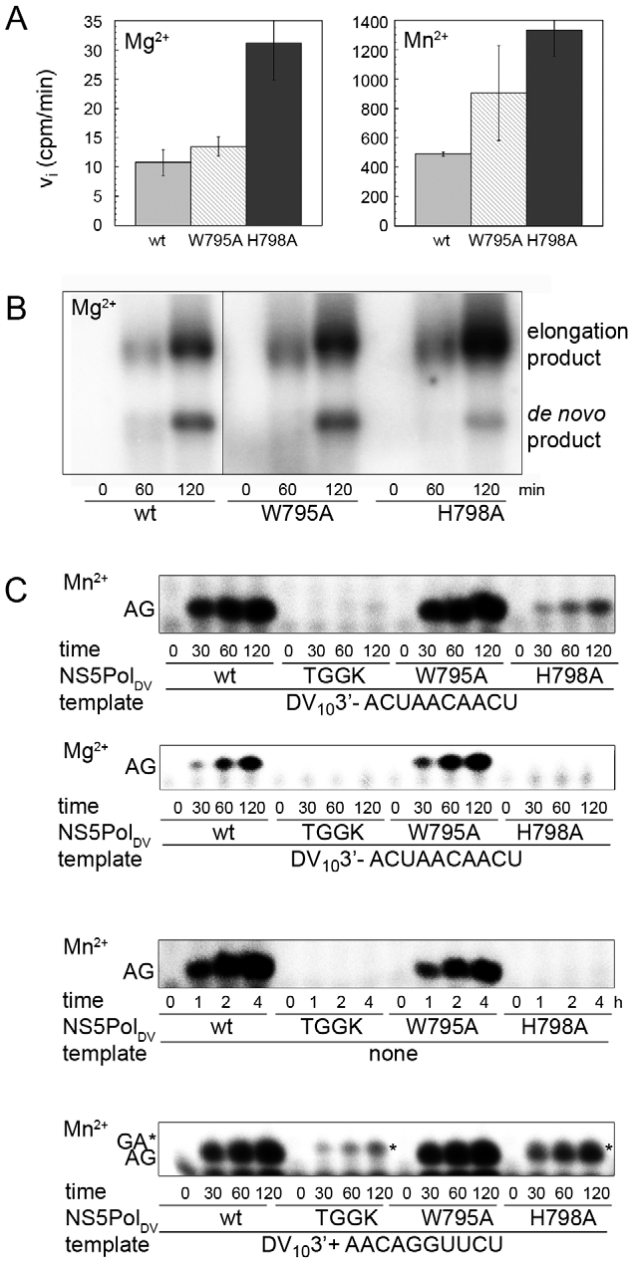
Role of NS5Pol_DV_ residues His798 and Trp795 as initiation platform. (**A**) Activity of wt NS5Pol_DV_ and its mutants W795A and H798A was determined on a specific minigenomic template. Reaction mixtures in the presence of [^3^H]-UTP were prepared as given in Materials and Methods. Initial velocities in cpm/min determined using filter-binding assays and liquid scintillation counting are compared in the presence of Mg^2+^ (left panel) and Mn^2+^ (right panel). (**B**) Reaction kinetics of wt NS5Pol_DV_ and its mutants W795A and H798A on the minigenomic template were analyzed on an agarose-formaldehyde gel. Reaction mixtures in the presence of [α-^32^P]-UTP and Mg^2+^ ions were prepared as given in Materials and Methods. Product bands are labeled on the right sight of the gel. (**C**) *De novo* initiation of wt NS5Pol_DV_, deletion mutant TGGK and mutants W795A and H798A was followed using either DV_10_3′- (1 µM in the presence of Mn^2+^ and 10 µM in the presence of Mg^2+^), in the absence of a template or 1 µM DV_10_3′+ (from top to bottom as indicated). Reaction mixtures contained 2 mM MnCl_2_ or 5 mM MgCl_2_ as indicated, 500 nM enzyme, 500 µM of ATP, and 100 µM GTP (containing αGTP). Reactions were started by addition of catalytic ions and samples were taken at given time points. Identities of labeled product bands are given on the left side of the gels. pppGA internal *de novo* initiation product on DV_10_3′+ is labeled by an asterisk.

*De novo* RNA synthesis initiation by wt NS5Pol_DV_ and the TGGK mutant were then tested on DV_10_3′-, in the absence of a template and on DV_10_3′+ using Mn^2+^ as the catalytic ion, ATP and GTP containing αGTP. Figure 5C (panel 1) shows that in contrast to wt NS5Pol_DV_, NS5Pol_DV_ TGGK is not able to catalyze *de novo* initiation on DV_10_3′-. Secondly, NS5Pol_DV_ TGGK does not catalyze pppAG formation without template (panel 2). In contrast, it is able to catalyze *de novo* initiation on DV_10_3′+ presenting ca. 32% of wt activity (panel 3). In order to understand this apparent contradiction, we used γATP instead of αGTP as radioactive NTP. It became clear that NS5Pol_DV_ TGGK was unable to generate the pppAG primer product (panel 4). We conclude that the product observed with αGTP corresponds to pppGA formed by internal *de novo* initiation being only possible on DV_10_3′+. When using Mg^2+^ as catalytic ion again we did not observe formation of the *de novo* RNA synthesis initiation product pppAG on either template (for DV_10_3′- see below Figure 6B).

We conclude that NS5Pol_DV_ TGGK is unable to pre-form the ATP-specific priming site necessary for *de novo* RNA synthesis initiation at the very 3′-end. The predicted priming loop plays indeed an essential role in providing the correct priming site. We explain the increased activity of NS5Pol_DV_ TGGK on minigenomic RNA templates by its increased propensity to catalyze back-priming due its more accessible catalytic site, *i.e.* to harbor the minigenome in different hairpin conformations allowing 3′ elongation.

### Residue H798 and not W795 is important for ATP-specific *de novo* initiation

Two aromatic residues, W795 and H798, within the priming loop were proposed to play a particular role in providing an initiation platform to which the base of the priming ATP could establish a stacking interaction [16]. Residue W795 was given special attention because it was found near the triphosphate moiety of a 3′-dGTP bound to NS5Pol_DV_ [16]. In addition, this tryptophan was better placed than the histidine for stacking a priming ATP in two models of *de novo* RNA synthesis initiation complexes of NS5Pol_DV_ and NS5Pol_WNV_ [16], [22]. We generated two mutants of NS5Pol_DV_, W795A and H798A. Overall correct folding of the purified recombinant mutants was equally verified by a fluorescent thermal shift assay giving T_m_ values corresponding to the wt protein (wt NS5Pol_DV_ T_m_ 49.0°C ± 0.5°C, W795A mutant T_m_ 48.6°C ± 0.6°C, H798A mutant T_m_ 48.1°C ± 0.04°C).

The RNA initiation and elongation activities of wt NS5Pol_DV_ and the W795A and H798A mutants were tested using the minigenomic RNA template and either Mg^2+^ or Mn^2+^ as catalytic ions (Figure 6A). In both cases the H798A mutant shows an increased activity on this template whereas W795A shows a similar overall activity compared to wt NS5Pol_DV_. Figure 6B shows the analysis of the reaction products on a denaturing agarose-formaldehyde gel. The W795A mutant behaves indeed like wt NS5Pol_DV_, the percentage of the *de novo* RNA synthesis initiation product of template size is unchanged. In contrast the H798A mutant generates considerably less *de novo* RNA synthesis product whereas the yield of RNA elongation products is higher.

We then compared the capacities of wt and all mutant NS5Pol_DV_ proteins to catalyze *de novo* RNA synthesis initiation on DV_10_3′-, without template and on DV_10_3′+ using Mn^2+^ as catalytic ion (Figure 6C panels 1, 3 and 4). Indeed, the H798A mutant is considerably less capable of correct *de novo* RNA synthesis initiation than wt NS5Pol_DV_ whereas W795A behaves as wt NS5Pol_DV_. Note that the product formed by NS5Pol_DV_ TGGK on DV_10_3′+ (panel 4) corresponds to pppGA generated by internal RNA synthesis initiation (see also Figure 5C); and therefore part of the product formed by the H798A mutant may correspond to pppGA. When Mg^2+^ is used on both templates, the same results are obtained (Figure 6C panel 2 for template DV_10_3′-). We thus conclude that residue H798 is essential for the formation of the correct ATP-specific priming site and may act as a priming platform.

## Discussion

In this study, we present evidence that the dengue virus NS5 polymerase domain (NS5Pol_DV_) alone is responsible for maintenance of A and U as first and last nucleotides of the DV genome, respectively. NS5Pol_DV_ was used instead of full-length NS5 in the frame of this study in order to avoid any interference of the RNA-binding, NTP-binding, or enzymatic activities of the N-terminal domain of NS5. We report that NS5Pol_DV_ is endowed with several structural and mechanistic features converging to the specific *de novo* synthesis and elongation of the correct ATP-initiated primer even on templates that lack the correct corresponding U at the 3′-end. The first and last nucleotides of the genome are strictly conserved in the genus *Flavivirus* thus the results presented here may apply to the entire genus.

**Figure 7:**
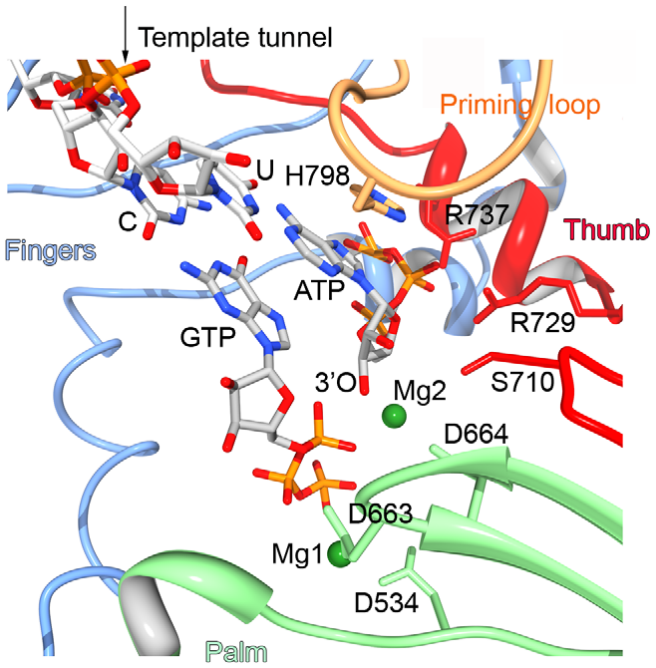
*De novo* initiation model of NS5Pol_DV_ in complex with RNA template UUCU (3′-end of DV genome), ATP, GTP and catalytic Mg^2+^ ions. The model was generated as explained in Materials and Methods. Fingers, thumb and palm subdomains are colored as in Figure 5A. Only the 3′-end CU of the RNA template is shown, it base pairs the initiating ATP and the second nucleotide GTP. The triphosphate of ATP contacts residues S710, R729 and R737 of the thumb subdomain motif E. The 3′-O atom of the ribose of ATP lies near the α-phosphate of GTP, which is coordinated to Mg1 bound to D534 of motif A and D663 of motif C. Mg2 is coordinated to the leaving pyrophosphate of GTP. The proposed priming platform H798 stacks to adenine base of the priming ATP.

We demonstrate the generation of a dinucleotide primer pppAG on both genomic and antigenomic RNA templates. We have previously observed the production of such dinucleotide primer on homopolymeric templates [14]. In the following step pppAG(A/U) trinucleotides are formed before processive RNA elongation occurs. During the latter, NS5Pol_DV_ continues RNA synthesis to the very end of the template. We do not know if di- and tri-nucleotide primers as detected in the reaction, originate from a slow but processive RNA synthesis reaction, or are actually released from the complex and re-used by the polymerase acting in a distributive RNA synthesis mode. We also show that the pppAG primer is effectively elongated in the presence of Mg^2+^ or Mn^2+^ and the correct template. Thus, after initial phosphodiester bond synthesis, the pppAG primer is aligned at the correct position in order to be elongated. The efficient use of the short primer pppAG reported here is in apparent contrast to the inefficient use of 5′-OH-AG dinucleotide previously reported [13], [30]. The 5′-triphosphate moiety of the chemically synthesized pppAG primer is most probably an important binding determinant allowing efficient elongation (see discussion of the proposed *de novo* initiation complex Figure 7).

We then demonstrate that in its *de novo* RNA synthesis initiation state NS5Pol_DV_ contains a built-in ATP-specific priming site. Major structural elements of NS5Pol_DV_ contributing to this site reside within residues T794 to A799. Their deletion forces NS5Pol_DV_ to initiate *de novo* RNA synthesis internal to the template using GTP as the first nucleotide (Figure 5C panel 1) and to perform primer-dependent RNA synthesis (Figure 5B). In analogy to the structure of HCV NS5B in complex with a nucleotide in its priming site [31] and because of the amino acid conservation observed within a larger group of *de novo* RdRps [25], we expect that NS5Pol_DV_ residues R472 (RdRp catalytic motif F3, see [14]) as well as S710 and R729 (motif E) are involved in triphosphate binding. This might explain why *de novo* RNA synthesis initiation by the loop-deleted mutant is still possible, albeit internal to the template. We conclude that indeed the T794-A799 loop plays a major role both in correct *de novo* initiation and in shaping the priming site. Within the priming loop, residue H798 is essential for primer synthesis (Figure 6). We propose that H798 provides the initiation platform against which the priming nucleotide ATP is stacked. Using the structure of the *de novo* initiation complex of the RdRp of bacteriophage φ6 [23] as a starting point, we generated a model of the initiation complex of DV serotype 2 RdRp in complex with the 3′- end of the genome UUCU and both ATP and GTP as first and second nucleotide, respectively (Figure 7). In this model, the triphosphate moiety of ATP indeed interacts with residues S710, R729 and R737 of the thumb subdomain of NS5Pol_DV_. The aromatic ring of H798 stacks the adenine nucleobase of ATP in a similar position to a φ6 RdRp tyrosine residue against which the guanine nucleobase of its priming GTP is stacked. In several protein complex structures histidine has been shown to bind an adenine nucleobase by stacking interactions [32]. Nevertheless, histidine does not seem to provide any specificity towards adenine versus guanine [33]. Our model does not propose any obvious specific interaction with the adenine base. This might be due to the fact that the structure of NS5Pol_DV_ has been captured in a pre-initiation state. In this state, motif F, which provides the upper part of the NTP entry tunnel in the active initiation and elongation conformation of viral RdRps, is not yet correctly positioned [34]. The fine characterization of the ATP-specific built-in priming site of NS5Pol_DV_ awaits the crystal structure of a *de novo* RNA synthesis initiation complex.

**Figure 8:**
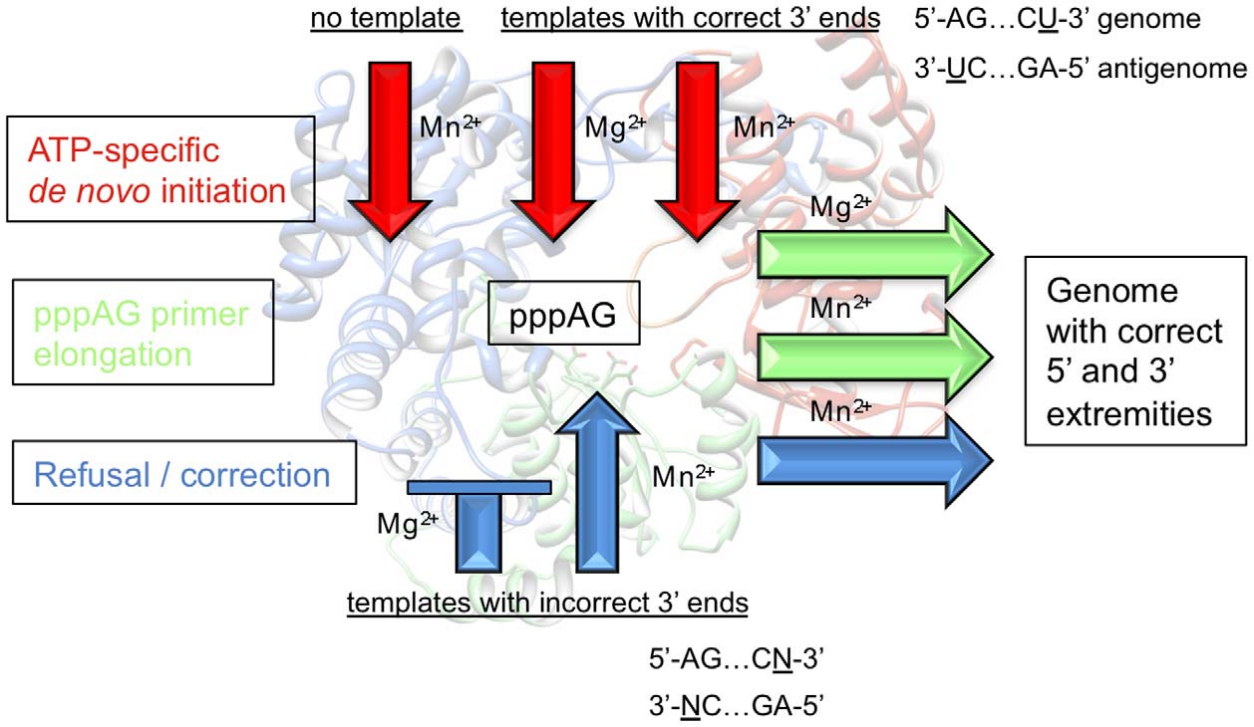
Dengue virus RdRp conserves the correct 5′- and 3′-ends of the genome. DV RdRp conducts strict ATP-specific *de novo* initiation in the absence of a template and in the presence of the correct template using the indicated catalytic ions Mn^2+^ or Mg^2+^. The pppAG primer is then elongated. When DV RdRp encounters templates with incorrect 3′-end nucleotides it refuses *de novo* initiation (when Mg^2+^ is present) or corrects the error by preferentially generating and elongating pppAG (using Mn^2+^ as catalytic ion). The structure of the DV2 RdRp domain is shown in the background.

We provide a mechanistic basis for the conservation of nucleotides A and U as the first and last nucleotides of the DV genome, respectively. Figure 8 summarizes the different levels of control that ensure ATP-specific *de novo* RNA synthesis initiation. Firstly, it generates and elongates the *bona fide* pppAG primer (red arrows and green arrows on the right). Even in the absence of any template and in the presence of Mn^2+^ (Figure 8 left red arrow) NS5Pol_DV_ is able to exclusively synthesize the pppAG primer (Figure 2B and C, Figure 3A and C). Note that we have also observed pppAG synthesis by full-length NS5 in the absence of a template (not shown). Since a sufficiently high Mn^2+^ concentration is present in the cell (0.1 µM to 40 µM Mn^2+^ in blood, brain, and other tissues [35]), NS5 in the replication complex might already be loaded with pppAG and thus be ready to elongate pppAG on the viral template. The same pppAG primer is preferentially synthesized in the presence of the correct template irrespective of the metal ion present at the polymerase active site (Figure 8 right red arrows, Figure 2A and B, Figure 3). In the presence of Mg^2+^, NS5Pol_DV_ supports neither formation nor elongation of pppAG on incorrect templates (Figure 8 blue blocked arrow, Figure 4B). In the presence of Mn^2+^, NS5Pol_DV_ is able to synthesize cognate dinucleotides on incorrect templates (Figure 2C), but in the presence of all nucleotides and all templates (a probably biased and more unfavorable set-up compared to the situation in the replication complex *in vivo*), pppAG is still a major product (Figure 3C). Remarkably, the pppAG/Mn^2+^-loaded polymerase is able to mismatch and extend pppAG in order to restore the correct 5′-end (Figure 8 blue arrows, Figure 4). The selective extension reaction thus refrains synthesis of incorrect RNAs that could occur in the presence of incorrect templates. All these reactions converge to the formation of pppAG and the conservation of A as the starting nucleotide at the 5′-end of viral genomic and antigenomic RNAs. Note that the mechanistic basis of the conservation of the second nucleotide G is beyond the scope of this study. Preliminary results generated in our laboratory indicate that both template and polymerase are important to ensure the specific incorporation of a G as the second nucleotide (not shown).

Several ways of viral RNA genome maintenance and repair concerning terminal damage have been discussed [4], among others the generation of “non-templated” primers and the use of abortive transcripts as primers. Here we demonstrate that NS5Pol_DV_ uses these two mechanisms. Non-templated primers are generated only in the presence of Mn^2+^. Abortive transcripts are used as primers in the presence of either Mg^2+^ or Mn^2+^. A third mechanism observed here is the discrimination against an incorrect template in the presence of Mg^2+^. In addition, in the case that a 3′-end might be shortened, the correction upon *de novo* initiation should be preceded by the addition of (a) nucleotide(s) by the terminal transferase activity of NS5. This activity has also been listed as another way of repairing terminal damage of viral RNA genomes [4]. For NS5Pol_DV_ we have observed this activity before [14] and now again in the presence of Mn^2+^ (Figure 1B).

The DV polymerase endows several of the proposed mechanisms to maintain the correct 5′ and 3′-ends of the DV genome and antigenome. The ability of DV and WNV to restore a U at the very 3′-end of genomes with 3′-end deletions has been demonstrated [2], [36]. This observation is in accordance with the existence of an ATP-specific priming site in NS5Pol_DV_. Tilgner *et al.* [2], [36] reported the complete reversion of WNV replicon CA and CG 3′-ends to CU whereas CC was only partially reverted. Since we have not seen preferential *de novo* RNA synthesis initiation starting with GG in comparison to UG or CG (all three are possible in presence of Mn^2+^, Figure 2), this might be due to an intrinsic difference between DV and WNV RdRp or caused by different propensities of the erroneous templates to allow pppAG elongation. Indeed CA and CG 3′-ends allow pppAG elongation more readily than the CC 3′-end (Figure 4, two independent reactions were performed). Thus the CC 3′-end might therefore take longer to revert. Furthermore, Teramoto *et al*. [2], [36] observed the correction of the 5′-end from pppGAG to pppAG. Our work provides a mechanistic explanation for their observation.

The observation of non-templated pppAG formation in the presence of Mn^2+^ by a viral RdRp has not been reported before using recombinant RdRp assays. However, previous reports convey the occurrence of non-templated dinucleotide formation. RSV, a member of the ns-RNA virus family *Paramyxoviridae* restores the correct 5′-pppA although minireplicons did not encode the correct 3′-U [10]. The authors propose that RSV RdRp contains a built-in ATP-specific priming site and cite the observation that the RdRp of the related ns-RNA vesicular stomatitis virus (VSV, *Rhabdoviridae*) contains a specific ATP-binding site [37] as an argument in favor of their proposition. When VSV RdRp assays were carried out using recombinant RdRp in the presence of Mg^2+^, non-templated 5′-initiation was not observed [6]. There is either the possibility that RSV and VSV belong to two different ns -RNA viral families and thus developed different strategies or, in analogy to our results that their RdRps use Mn^2+^ to correctly initiate RNA synthesis on erroneous templates as observed for NS5Pol_DV_ here. It is generally believed that Mg^2+^ is the activating cofactor of polymerases *in vivo* because viral RdRp properties observed with Mg^2+^ *in vitro* are more consistent with properties observed biologically. A second reason for giving the preference to Mg^2+^ is its cellular abundance in comparison to Mn^2+^ (*i.e.*, 0.5 mM free Mg^2+^ versus 0.7 µM free Mn^2+^ in rat hepatocytes [38], [39] and 0.1 µM–40 µM Mn^2+^ in blood, brain and other tissues [35] versus 0.2 to 0.7 mM Mg^2+^ in human blood [40]. Nevertheless, some events especially involved in correct and efficient *de novo* RNA synthesis initiation may require the specific use of Mn^2+^ by viral RdRps under physiological conditions (our study and [10], [36], [41], [42]).

The pppAG primer synthesis by the DV RdRp can be considered as the first line of control of the conservation of *Flavivirus* genome and antigenome ends. However, there might be other mechanisms to tighten the selection. The first one could be the base pairing of the genome ends maintaining specific RNA secondary structures, which are necessary to recruit the replication machinery. Computer simulations of such structures [43] indicate that the last U of the 3′-end of the genome may be unpaired or paired (structure I or II, respectively in [43]). Thus, requested base pairing may exert selective pressure to keep a U at the end of the *Flavivirus* genome. Another selection level concerns only the 5′-end of the genome and is due to the counterselection of incorrect 5′-ends through the NS5 RNA-cap methyltransferase. Indeed, several crystal structures of the cap-dependent bi-functional methyltransferase domain of NS5 show that specific binding of the 5′-cap involves specific recognition of the first transcribed 5′-adenosine through its N1 position and residue Asn18 [44], [45]. Therefore, for the genomic strand, methylation at the cap *N^7^*-guanine and the subsequent 2′-*O* position of the first transcribed adenosine should be efficiently achieved only when ATP is the starting 5′-nucleotide. Finally, cap addition seems to involve 5′-ATP selectivity as well [20]. Collectively, we propose that the RdRp of flaviviruses is the first actor responsible for the conservation of the correct ends of their genome, and that other mechanisms such as genome cyclization and the specificity of guanylyltransferase and methyltransferase activites add to the selective pressure. These mechanisms of maintenance might also apply to other RNA virus genera with conserved genome ends and viral RdRps initiating RNA synthesis *de novo*.

## Materials and Methods

### RNA templates

DV_10_3′+ (5′-AACAGGUUCU-3′) and DV_10_3′- (5′-ACUAACAACU-3′) were synthesized at the RIBOXX GmbH Dresden and by Dharmacon. The templates are devoid of stable secondary structure when submitted to the Mfold server [46] (ΔG = 3.60 kcal/mol for DV_10_3′+ and no folding for DV_10_3′-).

### Large-scale chemical synthesis and purification of pppAG

#### Chemical synthesis of AG on solid support

Chemical synthesis of the diribonucleotide AG was performed on an ABI 394 synthesizer (Applied Biosystems) from commercially available (Link Technologies) long chain alkylamine controlled-pore glass (LCAA-CPG) solid support with a pore size of 1000 Å derivatized through the succinyl linker with 5′-*O*-dimethoxytrityl-2′-*O*-acetyl-*N^2^*-dimethylformamide guanosine. The dinucleotide AG was assembled at a 8-µmol scale in Twist oligonucleotide synthesis columns (8×1-µmol scale) (Glen research) using 5′-*O*-DMTr-2′-*O*-pivaloyloxymethyl-*N^6^*-phenoxyacetyladenosine]-3′-*O*-(*O*-cyanoethyl-*N,N*-diisopropyl-phosphoramidite (Chemgenes) and following a previously described procedure [47]. After assembly completion, the CPG beads in the eight columns were dried under a stream of argon. The beads were pooled, divided in two, and around 4 µmol AG transferred into two Twist oligonucleotide synthesis columns (size for 10-µmol scale). The 5′-functionalization of AG with triphosphate moiety was performed in parallel with both columns following previously described conditions [48].

#### 5′-triphosphorylation of solid-supported AG

A solution (8 ml) of 1 M diphenyl phosphite (1.6 ml) in dry pyridine (6.4 ml) was manually passed with a glass syringe through the columns containing AG still attached to the solid support and left to stand for 30 minutes at room temperature. After several washings, the oxidation solution containing imidazole (375 mg, 5 mmol) in *N,O*-bis-trimethylsilylacetamide (1 ml, 4.1 mmol), CH_3_CN (1.875 ml), CCl_4_ (1.875 ml) and triethylamine (0.25 ml) was added under argon and left to react for 5 h at 30°C. After washing and drying the support, the TBAPP solution (0.23 M, 2 ml) was applied to the column and left to react for 18 h at 30°C. The solution was removed and the support was washed with dry CH_3_CN (4×8 ml). Finally, the column was dried by 1-min argon flush.

#### Deprotection and release of solid-supported pppAG

A 0.1 M solution of 1,8-diazadicyclo-[5,4,0]undec-7-ene (DBU) (1.2 ml) in anhydrous CH_3_CN (6.8 ml) was applied to each column for 3 min. Subsequently, a 30% aqueous ammonia solution was applied to each column in three batches (6 ml, 4 ml, 2 ml) for 30 min each. The three ammonia fractions were collected in screw-capped glass vials and were left to react at 30°C for 1.5 h. The fully deprotected pppAG was transferred to 50 ml round-bottomed flasks and isopropylamine (15% of total volume) was added to the solutions. Then the mixtures were evaporated under reduced pressure at 30°C until the volumes were reduced to 0.5 ml. The residues were redissolved in water (1.5 ml), transferred to 2 ml Eppendorf-vials and then lyophilized.

#### Analysis and purification of pppAG by reverse-phase HPLC

Analytical and semi-preparative HPLC was performed on a Dionex DX 600 HPLC system equipped with reverse-phase columns (Nucleodur C_18_, 100 Å, 3 µm, 4.6×70 mm for analysis and Nucleodur C_18_, 100 Å, 7 µm, 8×125 mm for purification, Macherey Nagel). The following solvent system was used: 5% CH_3_CN in 50 mM TEAB buffer, pH 8 (buffer A) and 80% CH_3_CN in 50 mM TEAB buffer, pH 8 (buffer B). Flow rates were 1 ml.min^−1^ and 2 ml.min^−1^ for analysis and semi-preparative purposes, respectively. Elution was performed with a linear gradient of 0% to 10% buffer B in buffer A in 20 min. The fractions containing the pure pppAG were pooled in a 100 ml round-bottomed flask and were concentrated to a volume of 0.5 ml under reduced pressure at 30°C. The residue was coevaporated ten times with 2 ml of water. The residue was redissolved in 1.5 ml water, transferred to 2 ml Eppendorf-vials and lyophilized. MALDI-TOF mass spectra were recorded on a Voyager-DE spectrometer (Perseptive Biosystems, USA) using a 10∶1 (m/m) mixture of 2,4,6-trihydroxyacetophenone/ammonium citrate as a saturated solution in acetonitrile/water (1∶1, v/v) for the matrix. Analytical samples were mixed with the matrix in a 1∶5 (v/v) ratio, crystallized on a 100-well stainless steel plate and analyzed. UV quantitation of pppAG was performed on a Varian Cary 300 Bio UV/Visible spectrometer by measuring absorbance at 260 nm. Two µmol of pure pppAG were obtained corresponding to 25% total yield. Lyophilized aliquots of 100 nmol have been stored at −20°C for several months without any sign of degradation.

### Protein expression and purification

The gene coding for N-terminal His_6_-tagged NS5Pol_DV_ (serotype 2, New Guinea C) as defined in [14] cloned in a pQE30 plasmid was expressed in *E.coli* (Tuner (Novagen) or NEB Express (New England Biolabs)) cells carrying helper plasmid pRare2LacI (Novagen). Expression was carried out in Luria broth overnight at 17°C after induction with 50 µM IPTG, addition of 2% EtOH and a cold shock (2 h at 4°C). Sonication was done in 50 mM sodium phosphate lysis buffer, pH 7.5, 500 mM NaCl, 20% glycerol, 0.8% Igepal (10 ml of this lysis buffer for around 2 g cell pellet from 1l culture) in the presence of DNase I (22 µg/ml), 0.2 mM benzamidine, protease inhibitor cocktail (SIGMA), 5 mM β-mercaptoethanol and 1 mg/ml lysozyme after 30 min incubation at 4°C. After centrifugation the soluble fraction was incubated in batch with 2 ml TALON metal-affinity resin slurry (Clontech) for 40 min at 4°C. Protein bound to the beads was washed once with 10 volumes of sonication buffer containing 1 M NaCl and 10 mM imidazole and once with the former buffer without Igepal. Protein fractions were then eluted with sonication buffer containing 250 mM imidazole, no Igepal and 250 mM glycine. After dialysis into 10 mM Tris buffer, pH 7.5 containing 300 mM NaCl, 20% glycerol, 250 mM glycine and 1 mM DTT the protein was diluted with the same volume of this buffer without NaCl and loaded onto a HiTrap heparin column (GE Healthcare). Pure NS5Pol_DV_ was then eluted in a single peak applying a gradient from 150 mM to 1 M NaCl. Alternatively, gel filtration was used as a second purification step using a Superdex 75 HR 16/60 column (GE Healthcare) and the dialysis buffer. NS5Pol_DV_ was stored at −20°C at a concentration of 40 to 60 µM after a final extensive dialysis into 10 mM Tris buffer, pH 7.5 containing 300 mM NaCl, 40% glycerol and 1 mM DTT. Purity was higher than 98% as judged by SDS-PAGE.

### Mutant NS5Pol_DV_ genes and proteins

Mutant TGGK, W795A and H798A NS5Pol_DV_ expression plasmids were generated using the kit QuikChange (Stratagene). Protein expression and purification was done as for the wt protein. Analysis by gel filtration showed a single peak eluting at the same volume as wt NS5Pol_DV_.

### Determination of T_m_ values

Melting temperature (T_m_) values of wt and mutant NS5Pol_DV_ were determined using a thermofluor-based assay [49]. In 96-well thin-wall PCR plates 3.5 µl of a fluorescent dye (Sypro Orange, Molecular Probes, 714-fold diluted in H_2_O) was added to 21.5 µl protein solutions at a concentration of 0.5 or 1 mg/ml (6.7 or 13.4 µM) in storage buffer. Thermal denaturation of the proteins was followed by measuring fluorescence emission at 575 nm (excitation 490 nm). T_m_ values were calculated using GraphPad Prism software and the Boltzmann equation as in [49].

### *In vitro* RdRp assays on DV_10_3′+ and DV_10_3′-

Reactions were done in 50 mM HEPES buffer, pH 8.0 containing 10 mM KCl, 10 mM DTT and template, NS5Pol_DV_, non-labeled NTPs, and catalytic ions at final concentration as given in the figure legends. Radiolabeled [γ-^32^P]-ATP, [α-^32^P]-GTP, or [α-^32^P]-UTP was used at 0.4 µCi per µl reaction volume (3000 Ci/mmol, Perkin-Elmer). Reactions were started by addition of a mixture of HEPES buffer, KCl, catalytic ions and UTP and CTP when used (given in Figures). After given time points samples were taken and reactions stopped by adding an equal volume of formamide/EDTA gel-loading buffer. Reaction products were separated using sequencing gels of 20% acrylamide-bisacrylamide (19∶1), 7 M Urea with TTE buffer (89 mM Tris pH 8.0, 28 mM taurine (2-aminoethanesulfonic acid), 0.5 mM EDTA). RNA product bands were visualized using photo-stimulated plates and the Fluorescent Image Analyzer FLA3000 (Fuji) and quantified using Image Gauge (Fuji). The oligoG marker was produced as explained in [14].

### *In vitro* RdRp assays on minigenomic template

The minigenomic template was produced by *in vitro* transcription and tests carried out as described in [14].

Reactions analyzed by filter-binding and liquid scintillation counting contained 50 mM HEPES buffer, pH 8.0, 10 mM KCl, 10 mM DTT, 100 nM RNA template, 200 nM NS5Pol_DV_, 500 µM NTP except for UTP (4 µM), [^3^H]-UTP at 0.2 µCi/µl and either 5 mM MgCl_2_ or 2 mM MnCl_2_. Reactions were started by the addition of a mixture of HEPES, KCl, catalytic ions, CTP, and UTP. After 30, 60, 90, and 120 min 10-µl samples were taken and diluted into 50 µl of 100 mM EDTA, pH 8.0 to quench the reaction. Samples were then transferred onto a DEAE filter mat. Non-incorporated [^3^H]-UTP was removed by washing with 300 mM ammonium formate and the radioactively labeled product quantified in counts per minute (cpm) using liquid scintillation counting. Product formation was then plotted against time and initial velocities calculated in cpm/min.

Reactions analyzed on formaldehyde-agarose gels contained 50 mM HEPES buffer, pH 8.0, 10 mM KCl, 10 mM DTT, 100 nM RNA template, 200 nM NS5Pol_DV_, 500 µM NTP except for UTP (4 µM), [α-^32^P]-UTP at 0.4 µCi/µl, and 5 mM MgCl_2_. Reactions were started by a mixture of HEPES, KCl, MgCl_2_, CTP and UTP and stopped after 60 and 120 min by adding an equal volume of sample buffer (40 mM MOPS pH 7.0, 83.3% formamide, 2 M formaldehyde, 10 mM sodium acetate, 85 mM EDTA). Samples were denatured for 10 min at 70°C and 1/10 of loading buffer (50% glycerol, 10 mM EDTA, xylene cyanol and bromphenol) added. Samples were then analyzed on a 1.2% agarose-formaldehyde gel in 20 mM MOPS buffer pH 7.0, 5 mM sodium acetate, 1 mM EDTA. Gels were dried and RNA product bands visualized using photo-stimulated plates and the Fluorescent Image Analyzer FLA3000 (Fuji) and quantified using Image Gauge (Fuji).

### Modeling of the NS5Pol_DV_ initiation complex

A homology model of NS5Pol_DV_ serotype 2 strain New Guinea C was generated using the Swiss-model server [50] and the X-ray structure of NS5Pol_DV_ serotype 3 (PDB code 2J7W [16]). NS5Pol_DV_ and the RdRp of bacteriophage φ6 in complex with a template RNA strand and initiating NTPs (PDB code 1HI0) were then superimposed using the three catalytic aspartate residues of both proteins. The structural model of the initiation complex of NS5Pol_DV_ serotype 2 was then generated by changing the RNA template to UUCU (3′-end of the DV genome) and the initiating NTP to ATP, and by manually adapting the conformation of the priming loop using the UCSF Chimera software [51]. Subsequently using the same program the computed free energy of the model was minimized.
